# LKAFFNet: A Novel Large-Kernel Attention Feature Fusion Network for Land Cover Segmentation

**DOI:** 10.3390/s25010054

**Published:** 2024-12-25

**Authors:** Bochao Chen, An Tong, Yapeng Wang, Jie Zhang, Xu Yang, Sio-Kei Im

**Affiliations:** 1 Faculty of Applied Sciences, Macao Polytechnic University, Macao 999078, China; p2316212@mpu.edu.mo (B.C.); p2312157@mpu.edu.mo (A.T.); xuyang@mpu.edu.mo (X.Y.); 2 Macao Polytechnic University, Macao 999078, China; marcusim@mpu.edu.mo

**Keywords:** urban land use, deep learning, CNN, feature restoration, smart city, sustainable building

## Abstract

The accurate segmentation of land cover in high-resolution remote sensing imagery is crucial for applications such as urban planning, environmental monitoring, and disaster management. However, traditional convolutional neural networks (CNNs) struggle to balance fine-grained local detail with large-scale contextual information. To tackle these challenges, we combine large-kernel convolutions, attention mechanisms, and multi-scale feature fusion to form a novel LKAFFNet framework that introduces the following three key modules: LkResNet, which enhances feature extraction through parameterizable large-kernel convolutions; Large-Kernel Attention Aggregation (LKAA), integrating spatial and channel attention; and Channel Difference Features Shift Fusion (CDFSF), which enables efficient multi-scale feature fusion. Experimental comparisons demonstrate that LKAFFNet outperforms previous models on both the LandCover dataset and WHU Building dataset, particularly in cases with diverse scales. Specifically, it achieved a mIoU of 0.8155 on the LandCover dataset and 0.9326 on the WHU Building dataset. These findings suggest that LKAFFNet significantly improves land cover segmentation performance, offering a more effective tool for remote sensing applications.

## 1. Introduction

The semantic segmentation of remote sensing imagery, classifying each pixel to identify land cover types, plays a crucial role in various applications, including urban planning, environmental monitoring, agriculture, and disaster management. Remote sensing images are characterized by complex, multi-scale features with substantial spatial variations, making segmentation particularly challenging. These complexities are further compounded by the need to balance global contextual information with fine-grained local details, especially in high-resolution images.

Remote sensing image classification is a crucial research direction in the fields of geographic information science and environmental monitoring. The evolution of methodologies in this domain has spanned multiple stages, from traditional statistical models to modern machine-learning approaches. Each method has demonstrated specific advantages in particular contexts, collectively laying a robust foundation for continuous technological advancements.

In previous studies, statistical methods dominated remote sensing classification. Among these, the Maximum Likelihood Classification (MLC) [1] approach, as one of the most classic statistical methods, assumes that the spectral characteristics of different classes follow a normal distribution. By calculating the conditional probability of each pixel belonging to a specific class, MLC assigns the pixel to the class with the highest probability. Supported by solid statistical theory, MLC is particularly effective in scenarios where spectral differences between classes are pronounced. However, MLC’s performance declines significantly when the actual data distribution deviates from the normality assumption. Additionally, its high computational complexity and limited adaptability in high-dimensional or multi-class scenarios further restrict its applications. In contrast, the Minimum Distance Classifier (MDC) [2] assigns pixels to the nearest class by calculating the Euclidean distance between the pixel and class centroids, while simple and computationally efficient, MDC disregards the shape and statistical properties of class distributions, leading to reduced accuracy in cases of overlapping spectral features or complex distributions. Similarly, Bayesian classifiers, rooted in statistical principles, leverage both prior and posterior probabilities, showing promising results in small-sample scenarios. Nevertheless, their performance heavily depends on the accuracy of prior knowledge and may introduce biases when class distributions are imbalanced.

With the increasing diversity and resolution of remote sensing data, rule-based classification methods have been gaining prominence. Unlike statistical methods, these approaches incorporate expert knowledge to explicitly encode spectral, spatial, and textural characteristics into classification rules. For instance, rule-set [3] classification utilizes predefined spectral or morphological criteria, making it particularly effective for domain-specific tasks such as land-use classification. Although highly interpretable, these methods are constrained by their reliance on expert-defined rules, limiting their generalizability to complex or dynamically changing environments. In comparison, Object-Based Image Analysis (OBIA) [4] segments images into objects and classifies them based on spectral, shape, and spatial relationships. By addressing the “salt-and-pepper” effect prevalent in pixel-level classification, OBIA is particularly suited for high-resolution imagery, producing more coherent results. However, its performance is highly sensitive to segmentation quality, and the parameterization of the segmentation process adds operational complexity.

To enhance classification performance further, feature transformation techniques have been increasingly integrated into remote sensing studies. These methods extract critical features or enhance separability, thereby improving classification efficiency and accuracy. For example, Principal Component Analysis (PCA) [5], a classic dimensionality reduction technique, linearly transforms data to extract major components, reducing redundancy and improving computational efficiency. However, the features extracted by PCA often lack physical interpretability, posing challenges for practical applications. Wavelet transform, another notable method, enables multi-scale analysis to capture both local and global features, excelling in the classification of objects with rich textural characteristics. Despite its potential to enrich classification information, the complexity of wavelet transform and its sensitivity to parameter selection may hinder its widespread practical adoption.

In the late 20th century, traditional machine learning methods began gaining traction in remote sensing image classification, offering significant advantages over statistical models in handling nonlinearity and adapting to complex land cover scenarios. Support Vector Machines (SVM) [6], for instance, achieve optimal class separation by identifying the best hyperplane in high-dimensional feature spaces. SVM is particularly effective in small-sample scenarios, delivering high classification accuracy in contexts with clear class boundaries. However, its performance is heavily dependent on kernel function selection and hyperparameter tuning, with computational complexity becoming a bottleneck for large-scale datasets. Random Forest (RF) [7], an ensemble learning method based on decision trees, demonstrates robust performance and high-dimensional data processing capabilities by aggregating multiple weak classifiers. While RF excels in heterogeneous land cover classification, it is sensitive to class imbalance, which can reduce accuracy for minority classes. Similarly, the K-Nearest Neighbors (KNN) [8] algorithm classifies pixels based on the proximity of the K nearest samples. Although intuitive and training-free, KNN is susceptible to noise and performs poorly with high-dimensional data, with relatively low classification efficiency. The Gaussian Mixture Model (GMM) [9], a probabilistic approach that assumes class distributions as mixtures of Gaussian distributions, can address overlapping class distributions but is highly dependent on initial parameters and data distribution, limiting its effectiveness in high-dimensional settings.

In summary, remote sensing classification techniques have evolved through a continuous accumulation of theoretical and practical advancements, from traditional statistical methods to machine learning. While these methods exhibit unique strengths in terms of classification accuracy, computational efficiency, and applicability, they also reveal challenges such as dependency on assumptions, parameter optimization complexity, and class imbalance issues. These approaches remain valuable for well-defined scenarios, small-sample tasks, and specific feature extraction applications. Future research should focus on enhancing the performance of these methods, incorporating emerging technologies such as deep learning, and extending their adaptability to complex and dynamic environments. This integration will offer new avenues for the theoretical and practical development of remote sensing image classification.

In the last decade, deep learning techniques have been extensively utilized in areas such as safety monitoring [10], handwritten digit recognition [11], human action [12] recognition, financial trading [13], remote image [14,15,16,17,18,19] processing and image recognition [20,21,22], among others [23]. Consequently, numerous researchers have explored the application of deep learning methods for analyzing remote sensing imagery [24,25]. These algorithms demonstrate superior performance compared to traditional machine learning approaches in identifying land cover types [26,27]. Typically, deep learning algorithms rely on CNNs for image [28] classification tasks. Traditional CNNs, such as U-Net [29], DeepLabv3+ [30], and PSPNet [31], have demonstrated strong performance in segmentation tasks. However, they face limitations in processing diverse spatial scales and complex contextual relationships inherent in remote sensing imagery. Models with fixed kernel sizes are particularly challenged when capturing both large-scale contextual information and small-scale object details. As a result, segmenting complex scenes with irregularly shaped objects or unclear boundaries remains difficult.

To address these issues, researchers have developed methods that integrate attention mechanisms and multi-scale feature extraction. Notable examples include F3Net [32] (Feature Filtering Fusing Network for Change Detection of Remote Sensing Images), which focuses on change detection, and CDFFNet [33] (Cross Dimensional Feature Fusion Network for Urban Land Use Detection), which enhances feature fusion across different dimensions. The DFFAN [34] (Dual Function Feature Aggregation Network for Semantic Segmentation of Land Cover) and MSFGNet [35] (Multi-Scale Features Gathering Network for Change Detection of Remote Sensing Images) are other state-of-the-art approaches that utilize multi-scale feature gathering and dual-function aggregation for improved segmentation in remote sensing imagery.

Recent studies have further highlighted the importance of integrating multi-scale aggregation and context-awareness to overcome the inherent challenges in remote sensing imagery. For instance, the gated multi-scale aggregation proposed in, Ref. [36] has significantly improved scene segmentation performance by better capturing complex contextual relationships. Similarly, approaches like PointFlow [37] have demonstrated the utility of point-based flow modeling for aerial image segmentation. Moreover, reviews such as [38] have comprehensively outlined advancements in change detection methodologies over the last decade, emphasizing the role of foundation models in multi-temporal remote sensing analyses, as discussed in ChangeN2 [39].

Recent efforts, such as the Large-Kernel Convolution [40] Application for Land Cover Change Detection, have also explored the benefits of larger convolutional [41] kernels in capturing broad contextual information while maintaining the precision needed for boundary delineation. These methods, however, face challenges when dealing with the highly diverse spatial layouts and spectral complexities of remote sensing data.

To address these limitations, this study proposes the Large Kernel Attention Aggregation (LKAA) module as a key component of LKAFFNet, designed to enhance the feature representation capability of the network. The core of its design is to optimize feature extraction by combining spatial attention and channel attention mechanisms. Given the complex spatial layout and significant spectral differences of land cover in remote sensing images, the LKAA module adaptively focuses on different regions and channels of features. This dynamic adjustment ensures that the model captures key land cover information in multi-scale scenes more accurately, thereby improving segmentation accuracy and robustness.

The LKAA module operates in two main stages: processing of the spatial attention mechanism and processing of the channel attention mechanism. These two mechanisms enhance and optimize the input feature map from the spatial and channel dimensions, respectively. Compared to previous methods that apply large kernel convolutions and multi-scale fusion, the LKAA module distinguishes itself by its explicit integration of dual attention mechanisms tailored to the challenges of remote sensing imagery, including complex spatial layouts and diverse land cover characteristics.

In response to these limitations, we propose LKAFFNet (Large-Kernel Attention Feature Fusion Network), a novel segmentation framework that combines large kernel convolutions, attention mechanisms, and multi-scale feature fusion. LKAFFNet introduces three core modules:

- Large-Kernel ResNet (LkResNet), which enhances multi-scale feature extraction using parameterizable large-kernel convolutions;
- Large-Kernel Attention Aggregation (LKAA), which combines spatial and channel attention for better feature representation;
- Channel Difference Features Shift Fusion (CDFSF), which efficiently fuses features across scales.

The input remote sensing imagery is first processed by the Large-Kernel ResNet (LkResNet) backbone, which extracts hierarchical and multi-scale feature representations. These features are then fed into the Large-Kernel Attention Aggregation (LKAA) module, where both channel-wise and spatial attentions are combined to highlight discriminative information. Meanwhile, the Channel Difference Features Shift Fusion (CDFSF) module aligns and fuses features across scales, ensuring that both global context and local detail are effectively preserved. Finally, the aggregated and refined features are upsampled to the original resolution, generating the final segmentation map. By integrating large-kernel convolutions, attentive aggregation, and cross-scale feature fusion, enables LKAFFNet to achieve robust and accurate results, even in complex scenarios with significant variations in scale and object boundaries.These innovations enable LKAFFNet to excel in segmenting both local details and global context, achieving superior performance in complex remote sensing scenarios.

Through extensive experiments, LKAFFNet outperforms existing methods such as DeepLabv3+ and DenseASPP, particularly in high-resolution datasets involving ambiguous boundaries and diverse scales. The structure of this paper is organized as follows: Section 2 describes the architecture of LKAFFNet; Section 3 presents the experimental setup and results; Section 4 discusses key findings and challenges; and Section 5 concludes with our contributions.

## 2. Proposed Method

This section introduces the general structure of LKAFFNet, followed by a detailed description of the functionalities of LkResNet, LKAA, and CDFSF.

### 2.1. Model Overview

**Figure 1:**
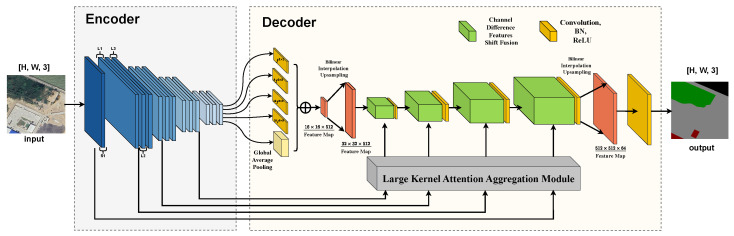
The structure of LKAFFNet.LKAFFNet is an efficient network for semantic segmentation, consisting of an encoder and a decoder. The encoder uses an improved LKResNet framework with large-kernel reparameterized convolutions for multi-scale feature extraction. The decoder includes a LKAA, which combines multi-scale large-kernel convolutions and Global Average Pooling to generate dynamic weights for feature fusion via self-attention. Additionally, the CDFSF module enhances channel coherence and strengthens interactions between high-frequency details and semantic information. By integrating multi-scale feature extraction, detail enhancement, and context aggregation, LKAFFNet achieves high segmentation accuracy.

The architecture of LKAFFNet is designed to address the challenges of multi-scale feature extraction and fusion in the semantic segmentation of remote sensing imagery. The design principle combines large-kernel convolutions with attention mechanisms to effectively capture the geometric features and contextual information of land covers at different scales. Below is a detailed introduction to the LKAFFNet modules: The overall structure of LKAFFNet is modular, with each component serving a specific role. Through feature extraction, fusion, and enhancement, the model progressively improves accuracy and efficiency in semantic segmentation tasks. The base layer consists of the LkResNet module, which is responsible for the multi-level feature extraction from input remote sensing images, utilizing large-kernel convolutions to expand the receptive field. The intermediate layers include the LKAA and CDFSF modules, which enhance feature representation through attention mechanisms and optimize multi-scale feature fusion, respectively. At the top layer, the ASPP module further aggregates multi-scale contextual information, providing a comprehensive semantic representation for the final segmentation output. The structure of LKAFFNet is shown in Figure 1.

The design goal of the network is to achieve the global perception of large-scale backgrounds while ensuring the precise segmentation of fine-grained land cover edges. When processing complex scenes in remote sensing imagery, LKAFFNet effectively aggregates information across different scales and addresses common segmentation challenges through the collaborative functioning of its various modules.

Mathematically, the overall process of the S1 encoder for the feature extraction can be represented as follows:

(1) $$Y=f_{1}^{3\times 3}\left(f_{1}^{1\times 1}\left(f_{1}^{3\times 3}\left(f_{1}^{RC}\left(f_{1}^{3\times 3}\left(x\right)\right)\right)\right)\right),$$

Among them, x represents input. *Y* represents output. $f_{1}^{RC}$ represents reparameterized convolution. $f_{1}^{1}$ represents 1×1 convolution. The settings for 3×3 are the same. All the activation functions are ReLU, which have been omitted.

The feature map is processed by the S1 encoder and sent to the L1 encoder for the next step of feature extraction. The S1 encoder is as follows:

(2) $$Y=f_{1}^{1\times 1}\left(f_{1}^{3\times 3}\left(f_{1}^{RC}\left(f_{1}^{1\times 1}\left(x\right)\right)\right)\right)⊕f_{1}^{1\times 1}\left(x\right),$$

The parameter situation is the same as above. Mathematically, the formula for the L2 encoder is as follows:

(3) $$Y=f_{1}^{3\times 3}\left(f_{1}^{1\times 1}\left(\mathrm{Se}(\mathrm{bn}\left(f_{1}^{RC}\left(f_{1}^{1\times 1}\left(x\right)\right)\right))\right)\right)⊕f_{1}^{1\times 1}\left(x\right),$$

The parameter situation is the same as above. It is worth noting that the BN layer does not have an activation function, while the activation function of the SE layer is sigmoid. The activation function of the 1×1 convolutional layer after the SE layer is GeLU , not ReLU .The structure of the L3 encoder and L2 encoder is similar, but the difference lies in the number of channels that change during processing, so they will not be listed here.

### 2.2. Large-Kernel ResNet

The Lkresnet module serves as the backbone of LKAFFNet, tasked with multi-level feature extraction. Its design is inspired by the traditional ResNet [42] (Residual Network) but incorporates significant innovations and optimizations, particularly in the enhancement of residual connections and the bottleneck module. The traditional ResNet relies on 3 × 3 convolutional kernels to process fine-grained features and uses residual connections to mitigate gradient vanishing in deep networks. In contrast, Lkresnet significantly improves upon these foundations by introducing parameterizable large-kernel convolutions, which strengthen the model’s ability to capture large-scale and global contextual information.

**Figure 2:**
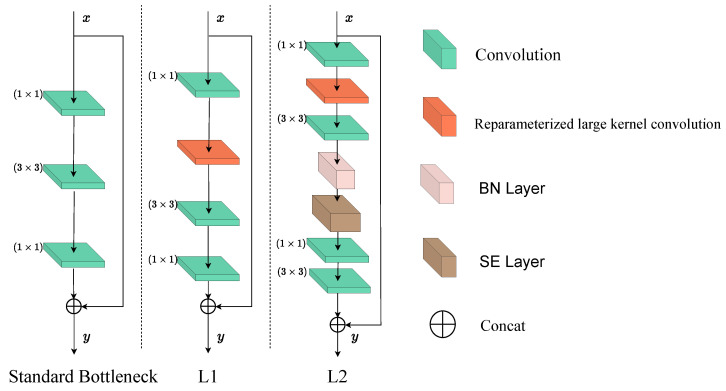
The figure includes the standard bottleneck structure and two improved modules (L1 and L2). The standard bottleneck structure consists of consecutive convolution operations, while the L1 module introduces reparameterized large-kernel convolution layers to enhance feature extraction capabilities. The L2 module further incorporates a BN Layer and attention-enhanced SE layers, with feature fusion achieved through concatenation. These designs aim to optimize network performance and improve feature representation. It should be noted that the structures of L3 and L2 are similar, with the difference being the number of channels, so they will not be repeated here.

Lkresnet’s residual [43] block differs from those in traditional ResNet. In the latter, the residual block is implemented by directly adding the input to the output after convolution, ensuring smooth gradient backpropagation. Lkresnet innovates on this classic design, particularly in the bottleneck module. Instead of stacking multiple 3 × 3 kernels for feature extraction, Lkresnet utilizes parameterizable large-kernels. The structure of the residual block is shown in Figure 2.

This figure illustrates the design and comparison of the standard bottleneck structure and its two improved variants (L1 and L2), each optimized for efficient feature extraction in deep learning. The standard bottleneck structure employs a classic 1 × 1 convolution for dimensionality reduction, followed by a 3 × 3 convolution for feature extraction, and another 1 × 1 convolution to restore channel dimensions. The output is obtained by adding the result to the input features via a skip connection. Although this design is computationally efficient, the limited receptive field of the intermediate 3 × 3 convolution kernel may hinder its ability to capture long-range dependencies in complex scenarios, thus constraining the model’s expressive power and robustness.

To address these limitations, the L1 structure introduces targeted improvements. It retains the fundamental workflow of the standard bottleneck structure but replaces the 3 × 3 convolution with a re-parameterized large-kernel convolution. The large kernel significantly expands the receptive field, enhancing the capacity for global information modeling. Meanwhile, the reparameterization technique improves training efficiency without increasing inference-phase computational overhead, enabling the model to extract high-quality features over a broader spatial scope.

Building on L1, the L2 structure further incorporates an attention mechanism (SE layer) to dynamically reweigh channel-wise features based on global information. This mechanism enhances the model’s ability to focus on critical features while suppressing irrelevant or redundant ones, thereby improving feature selectivity. Additionally, L2 employs concatenation rather than simple addition for the final output. This design enables the model to integrate features in more dimensions, enriching the feature representation. Compared to the standard bottleneck, L2 not only captures a larger receptive field but also processes interchannel information more effectively, making it particularly adept at handling complex tasks.

In summary, while the standard bottleneck structure is simple and efficient, its limited receptive field and restricted feature selection capabilities make it less suitable for complex scenarios. L1 improves feature modeling by expanding the receptive field through re-parameterized large-kernel convolutions, whereas L2 further optimizes channel-wise feature selectivity and multi-scale representation by integrating attention mechanisms and feature concatenation. These enhancements make the improved structures more suitable for tasks demanding high precision and generalization.

**Figure 3:**
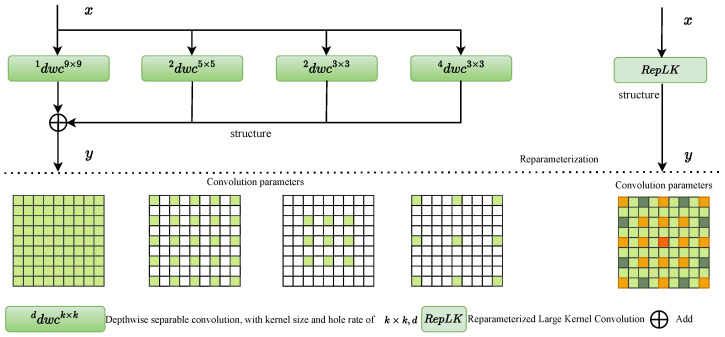
The structure of the reparameterizable large-kernel convolutions. The left part represents the original sequential convolution structure, where multiple depthwise convolutions with different kernel sizes and dilation rates are combined. The right part demonstrates the reparameterized structure (RepLK), where the equivalent convolution parameters are merged into a single large-kernel convolution. The bottom section shows the convolution parameter mapping process, highlighting how the parameters from sequential convolutions are consolidated into the reparameterized large-kernel convolution.

The introduction of parameterizable large-kernels is one of LkResNet’s core advancements. Unlike traditional small kernels, large kernels can capture contextual information over a broader range, effectively enhancing the network’s capacity to handle large-scale objects. In practice, Lkresnet employs 7 × 7 or even larger convolutional kernels, significantly expanding the receptive field and enabling the model to address both large-scale background information and fine-grained local features. Importantly, this large-kernel design employs reparameterization techniques that adjust parameters during training while optimizing to reduce computational load during inference. This approach not only enhances feature extraction efficiency but also reduces the model’s computational overhead. The structure of the parameterizable large kernels is shown in Figure 3.

In Lkresnet, the residual connections are not merely reused from traditional ResNet but are integrated with parameterizable large-kernels. In each bottleneck block, input features undergo multi-scale extraction via large-kernel convolutions before being added to the original input through residual summation. This process ensures the flow of deep features and enhances the network’s capacity to perceive large-scale and global information through multi-level convolutions. The modified residual connections effectively address the gradient vanishing issue while improving the model’s ability to capture global context.

To better capture information across different scales, Lkresnet incorporates dilated convolution on top of large-kernel convolutions. Dilated convolution expands the receptive field of the convolutional kernel without adding extra computational costs. Specifically, Lkresnet employs varying dilation rates, enabling the convolution operations to extract features over larger pixel ranges. For instance, smaller dilation rates are suitable for capturing fine-grained edge features, while larger dilation rates help capture large-scale global information.

This multi-scale feature extraction design ensures that LkResNet can handle both large-scale backgrounds and small-scale targets when processing complex remote sensing images. In scenarios common in remote sensing, such as multi-scale scenes, LkResNet achieves more precise segmentation through this mechanism. Analyzing the module structure reveals that each convolutional operation at different layers introduces varying dilation rates, further enhancing the model’s ability to extract and aggregate features across different scales.

Each convolution operation in LkResNet not only extends the receptive field but also enhances feature representation through batch normalization and nonlinear activation functions, such as ReLU. The introduction of batch normalization stabilizes the training process and mitigates the effects of internal covariate shift, while the ReLU activation function increases the network’s nonlinearity, enabling better fitting in complex scenarios.

Moreover, LkResNet’s multi-layer convolution design ensures a gradual refinement and enhancement of features from low to high levels. At lower levels of the network, the focus is on basic structures and local features, while deeper convolutional layers progressively capture more complex semantic information and global context. This bottom-up feature extraction approach provides high-quality feature inputs for subsequent attention and fusion modules.

The reparameterization technique in LkResNet significantly supports the application of large-kernel convolutions. By adjusting convolutional kernel parameters during training, reparameterization ensures that these kernels can be computed more efficiently during inference. This not only reduces resource consumption but also increases the execution speed of convolution operations. The incorporation of this technique allows LkResNet to enhance inference speed while maintaining accuracy when processing large-scale remote sensing images, which is critical for practical applications.

### 2.3. Large-Kernel Attention Aggregation Module

The LKAA module is a key component of LKAFFNet. The core of its design is to optimize the feature representation capability of the network by combining spatial attention and channel attention mechanisms. Given the complex spatial layout and significant spectral differences of land cover in remote sensing images, the model must adaptively focus on different regions and channels of features. The LKAA module dynamically adjusts the network’s attention to various spatial positions and feature channels, ensuring that the model accurately captures key land cover information in multi-scale scenes, thereby improving segmentation accuracy and robustness.

**Figure 4:**
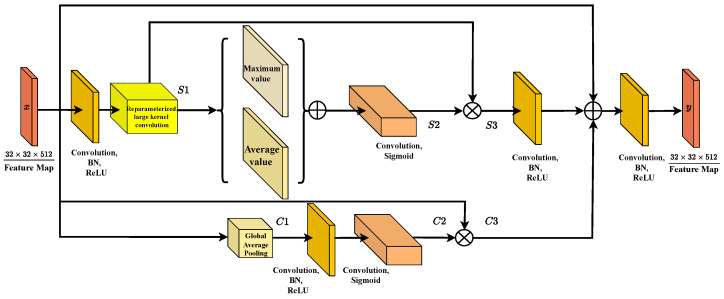
The LKAA architecture begins with reparameterized large-kernel convolution for feature extraction, followed by three stages (S1, S2, S3). In S1, global pooling and multi-scale feature fusion are combined with an attention mechanism that enhances input features via channel-wise importance weights. S2 and S3 process features using convolution, batch normalization, and activation functions. The final output is produced by fusing features through multiplication and sigmoid activation, leveraging hierarchical extraction, multi-scale fusion, and attention for improved performance.

The entire process of the LKAA module can be divided into the following two main stages: processing of spatial attention mechanism and processing of channel attention mechanism. These two mechanisms enhance and optimize the input feature map from the spatial and channel dimensions, respectively. The specific process of this module is shown in Figure 4.

The spatial attention mechanism enables the model to dynamically focus on salient regions in the feature map. Specifically, it analyzes the spatial distribution of feature maps to determine which pixels are more important for the current segmentation task while identifying those that can be ignored. The processing flow is as follows: Firstly, the input feature map undergoes GAP and GMP. These operations compress the original feature map into two lower-resolution maps, representing the average and maximum information of the features, respectively. This preserves the overall semantic information in the image, helping the network capture the global context. Next, the pooled feature maps are processed through convolutional layers to generate spatial attention maps. The purpose of this convolution is to further optimize and integrate spatial information, ensuring that the attention weight of each pixel is adjusted based on its relevance to the global context. The generated spatial attention map is multiplied with the original feature map, thereby enhancing the network’s attention to important regions while reducing its response to irrelevant regions. This operation ensures that the model prioritizes pixels with important features in complex scenes, such as building edges and road intersections in remote sensing images. Through this series of operations, the spatial attention mechanism achieves adaptive adjustment along the spatial dimension of the feature map, enabling the network to accurately capture the target area in the presence of complex geometric shapes and fuzzy boundaries, ultimately improving segmentation accuracy.

The channel attention mechanism is a supplement to the spatial attention mechanism, running on the channel dimension of feature maps. In multispectral or multi-source data, different channels typically represent different semantic information, some of which are crucial for specific tasks, while others may only contain noise. The goal of the channel attention mechanism is to dynamically evaluate the importance of each channel and enhance attention to effective channels while suppressing less important or redundant channels. Similar to the spatial attention mechanism, the channel attention mechanism first applies GAP and GMP to the input feature map. However, in this case, pooling operations are performed across channel dimensions, compressing the information of each channel into a single scalar. This extraction enables the network to evaluate the global semantic information of each channel. Next, the pooling features are passed through a two-layer fully connected layer, and the middle layer is processed through an activation function (ReLU) to generate a set of channel attention weights. These weights represent the network’s evaluation of each channel, with higher values indicating greater importance for the current task. The generated channel weights are fused with the original feature map through multiplication operations for each channel, thereby generating optimized feature maps along the channel dimension. This operation ensures that the model prioritizes the use of channels with strong representation capabilities, thereby improving overall segmentation performance. The channel attention mechanism dynamically adjusts the importance of each channel to ensure that the network intelligently selects representative feature channels while minimizing noise and redundant channel interference when processing complex multispectral remote sensing images. In summary, mathematically, the feature map Y can be represented by the following formula:

(4) $$Y=f_{1}^{1\times 1}\left(C_{3}⊕x⊕f_{1}^{1\times 1}\left(S_{3}\right)\right),$$

The core innovation of the LKAA module lies in the clever integration of spatial and channel attention mechanisms. This combination enables the model to perform dynamic feature optimization in two dimensions, thereby improving the accuracy of segmentation tasks. The spatial attention mechanism optimizes the feature map at the pixel level, allowing the model to prioritize salient regions and key boundaries in the image. Meanwhile, channel attention further optimizes features along the channel dimension to ensure that the model extracts the most representative feature information from different channels. Considering that Lkresnet already provides a rich set of multi-level functions, the LKAA module further integrates and enhances these functions through attention mechanisms, ensuring the optimal balance between global context and local details.

The advantage of the LKAA module is that although it introduces a dual level attention mechanism, its computational complexity is still relatively low, especially when combined with reparameterized large-kernel convolutions. This enables the LKAA module to maintain high accuracy while avoiding excessive computational costs, making it suitable for deployment in practical applications, especially for large-scale remote sensing data.

This dual-level attention mechanism endows the LKAA module with strong adaptability, enabling it to handle highly complex scenes with similar spectral features or significant geometric changes. Through the synergistic effect of spatial and channel attention, the LKAA module effectively enhances the model’s ability to distinguish multi-scale and multi-class targets. The LKAA module plays a crucial role in enhancing and optimizing the functionality of the overall architecture of LKAFFNet.

### 2.4. Channel Difference Features Shift Fusion

The CDFSF module focuses on the fusion of features at different scales. In remote sensing images, objects often exhibit significant size differences, such as buildings, roads, and forests, making the fusion of multi-scale features critical for enhancing segmentation accuracy. The CDFSF module effectively facilitates the recombination and integration of features across scales through feature transformation and channel fusion techniques.

**Figure 5:**
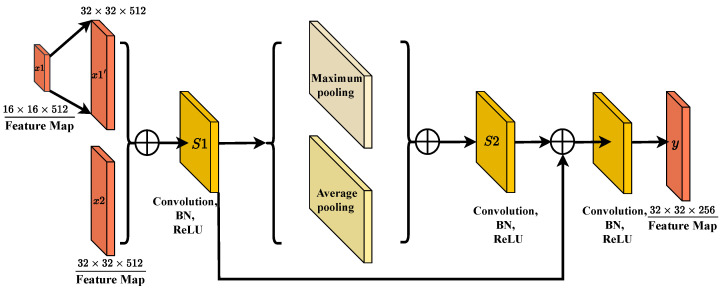
The CDFSF architecture processes feature maps (x1, x2) through convolutional layers with batch normalization and activation to extract features. It applies max and average pooling for multi-scale information, concatenates the results for feature fusion, and uses linear interpolation for spatial alignment. The fused features are processed to generate the final output (y), emphasizing efficient feature interaction and multi-scale representation for robustness.

By strategically shifting and fusing channel differences, CDFSF allows the model to capture varying object characteristics and spatial relationships, thereby enriching the representation of multi-scale features. This approach ensures that the model can effectively process and integrate information from diverse object scales, ultimately improving the precision and robustness of the segmentation outcomes. The structure of the CDFSF is shown in Figure 5.

In the CDFSF module, the input feature map x1 is first upsampled using bilinear interpolation to align its resolution with that of feature map x2. Subsequently, the two feature maps are element-wise added to achieve an initial fusion. This step ensures spatial consistency and semantic relevance of multi-scale features by aligning resolutions and performing pixel-wise addition. The fused feature map is then processed through a convolution layer, batch normalization, and ReLU activation to produce feature map s1. This operation leverages convolution to extract deep features, stabilizes the training process with batch normalization, and enhances the nonlinear representation capacity through ReLU activation.

Next, s1 is split into two branches, undergoing max pooling and average pooling, respectively, to capture local salient features and global statistical properties. Simultaneously, s1 is further processed by a 3 × 3 convolution layer followed by batch normalization and ReLU activation, generating feature map s2. This design effectively combines the pooling operations’ ability to extract local and global information with the 3 × 3 convolution’s capability to capture complex spatial relationships and semantic features. The feature maps s2 and s1 are then element-wise added for further feature refinement. By adopting this multi-branch fusion strategy, the module enhances feature diversity and robustness.

Finally, the fused feature map is passed through a 1 × 1 convolution layer, batch normalization, and ReLU activation to produce the final output feature map y. This step efficiently compresses channel information while further integrating multi-level feature representations. The advantages of this module are twofold: on one hand, it captures both salient and global statistical information through multi-scale feature alignment and multi-branch pooling; on the other hand, it ensures computational efficiency and expressive power through element-wise addition and lightweight convolution-based multi-stage fusion. This design makes the module well-suited for visual tasks that demand efficient and robust feature representation. In summary, mathematically, the feature map Y can be represented by the following formula:

(5) $$Y=f^{3\times 3}\left(S_{1}⊕S_{2}\right),$$

The strength of the CDFSF module lies in its ability to handle multi-layer features from different scales and enhance the model’s robustness to complex scenes through fusion operations. This capability enables LKAFFNet to maintain high segmentation accuracy in scenarios that encompass both large-scale backgrounds and small-scale targets.

## 3. Experiment and Result Analysis

This section provides a detailed description of the experimental results of each model on the urban land cover dataset. The results indicate that LKAFFNet outperforms other models.

### 3.1. Dataset

In this study, the LandCover.ai dataset [44] is utilized for the semantic segmentation experiments. The LandCover.ai dataset consists of images collected from a rural area in Poland, covering 216.27 square kilometers, with manual annotations for the following four object categories: buildings, forest, water, and roads. The original images in LandCover.ai were cropped to yield 10,674 images, each sized 512 × 512 pixels. These images were randomly divided into training, validation, and testing sets in a ratio of 0.7:0.15:0.15, resulting in 7470 images for training, 1602 images for validation, and 1602 images for testing.

**Figure 6:**
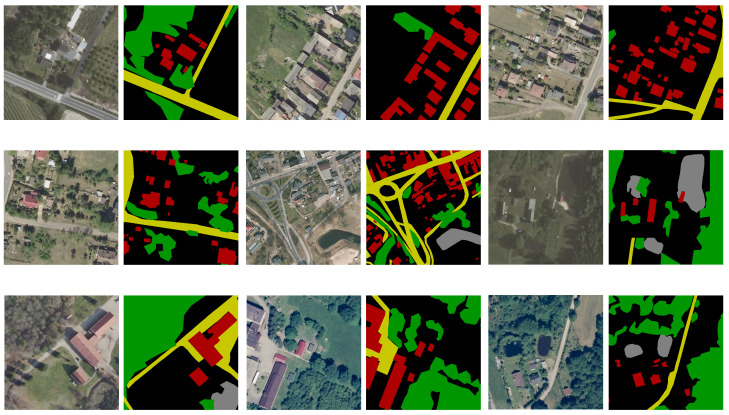
Sample images in the dataset. Red represents buildings, green denotes forest, gray indicates water bodies, yellow signifies roads, and black represents the background.

Figure 6 shows the cropped images from the dataset along with their corresponding ground truth. The left column displays the images, while the right column shows the segmentation results.

### 3.2. Experiment Setting and Training

In the experiments, the initial learning rate was set to 5 × 10^−5^, with a learning rate adjustment strategy based on cosine annealing. This learning rate decay strategy reduces the learning rate according to a cosine function throughout the training process. Specifically, the learning rate starts high and gradually decreases, reaching its minimum at the end of training. The mathematical formulation for the cosine annealing learning rate can be expressed as follows:

(6) $$\eta \left(t\right)=\eta_{min}+\frac{1}{2}\left(\eta_{max}-\eta_{min}\right)\left(1+\cos\left(\frac{T_{cur}}{T_{max}}\pi \right)\right),$$

where η(t) is the learning rate at time step *t*; $\eta_{min}$ is the minimum value of the learning rate; and $\eta_{max}$ is the maximum value of the learning rate, which is usually the initial learning rate. $T_{cur}$ is the current training generation number, and $T_{max}$ is the total number of generations.

The maximum number of iterations was set to 300. The cosine annealing period was configured as one-third of the total training iterations. Cross-entropy was used for loss calculation; in the PaddlePaddle framework, paddle.nn.CrossEntropyLoss integrates softmax and cross-entropy loss calculations internally, allowing for direct computation using logits and labels without manually applying softmax. Thus, the cross-entropy calculation can be simplified to the following:

(7) $$L=\frac{1}{N}\sum_{n=1}^{N}-y_{n}\log\left(p_{n}\right),$$

where $p_{n}$ is the probability distribution of sample *n* predicted by the model, and $y_{n}$ is the true label of sample *n*.

### 3.3. Ablation Experiments and Result Analysis

Ablation experiments were conducted to validate the contribution of each functional module. The experiments were divided into the following four groups: LKAFFNet (without LkResNet), which replaced LkResNet with ResNet50; LKAFFNet (without LKAA), which excluded the LKAA module; LKAFFNet (without CDFSF), which omitted the CDFSF module; and the standard LKAFFNet group. The experimental results for each group are shown in Table 1 and Table 2. Table 1 lists the overall performance metrics, including MPA, mIoU, and Kappa; Table 2 shows the intersection over union of each class and the F1 score of each class, representing the weighted average of all classes.

From Table 2 and Table 3, it is evident that the model’s segmentation performance is significantly impacted by these modules. The mIoU for the LKAFFNet (without LkResNet) group is 0.032 lower than that of the standard LKAFFNet group, with other metrics for the LKAFFNet groups (without LkResNet) also falling far below those of the standard LKAFFNet group. This is attributed to the LkResNet module’s use of parameterizable large-kernel convolutions, which reduce computational cost compared to using a single large kernel. Additionally, the use of multiple dilated convolutions allows for feature extraction at various scales, resulting in more comprehensive features and, consequently, improved performance.

Moreover, while both LKAA and CDFSF integrate features in distinct ways, they both enhance feature representation, leading to improved segmentation results. Overall, the impact of LKAA on the segmentation outcomes is greater than that of CDFSF.

Table 2 indicates that the prediction metrics for the background are higher than those for buildings and roads. This can be attributed to the fact that the percentage of pixels classified as background is greater than that of other categories; thus, for classes with a larger sample size, recognition accuracy is generally better.

Similarly, for the WHU Building Dataset (Table 4), the ablation experiments demonstrate consistent trends. The mIoU for the LKAFFNet (without LkResNet) group is 0.338 lower than that of the standard LKAFFNet group, again showing a notable decrease in performance across all metrics. The exclusion of the LKAA module also leads to a decline in the overall performance, as the module enhances the model’s ability to focus on critical features through attention mechanisms. Furthermore, the omission of the CDFSF module negatively affects the segmentation results. These results further confirm the robustness and generalizability of the LKAFFNet framework when applied to diverse datasets, highlighting the importance of each functional module in achieving superior performance.

### 3.4. Comparative Experiment and Result Analysis

To validate the performance of the LKAFFNet model in land cover detection, comparisons were made with models including DANet [45], DeepLabv3+ [30], DenseASPP [46], HRNet [47], LEDNet [48], OCNet [49], UPerNet [50], PSPNet [31], and UNet [29]. All models were tested under identical conditions, meaning they shared the same initial learning rate, momentum, and optimizer, and that they were trained on the same hardware. The training results for each model on the Landcover.ai dataset are summarized in Table 5, with IoU and F1_score metrics detailed in Table 6.

As shown in the table above, the proposed LKAFFNet employs re-parameterized convolutions, a design that significantly enhances both computational efficiency and performance. During the training phase, re-parameterized convolutions incorporate more complex structures, such as multi-branch architectures or larger convolutional kernels, to improve feature extraction capabilities. In the inference phase, these structures are simplified into standard convolutions, drastically reducing computational complexity.

When compared with other networks, LKAFFNet achieves superior performance while maintaining an extremely low computational cost of only 13.5 GFLOPs and a moderate parameter count of 14.9 M. Notably, networks such as HRNet (72.8 GFLOPs, 64.7 M), and OCNet (55.3 GFLOPs, 50.2 M) achieve competitive performance but require significantly higher computational resources and parameter storage. In contrast, lightweight networks like LEDNet (9.6 GFLOPs, 4.7 M) reduce computational costs and parameter count but compromise performance, achieving lower MPA and MIoU.

LKAFFNet strikes an optimal balance between performance and efficiency, outperforming all listed models with an MPA of 0.9501, MIoU of 0.8203, and Kappa of 0.9069, while maintaining a much lower computational burden compared to its more complex counterparts. Its parameter count of 14.9 M further demonstrates its lightweight design, offering an efficient yet powerful solution for real-world scenarios where both performance and resource efficiency are critical.

**Figure 7:**
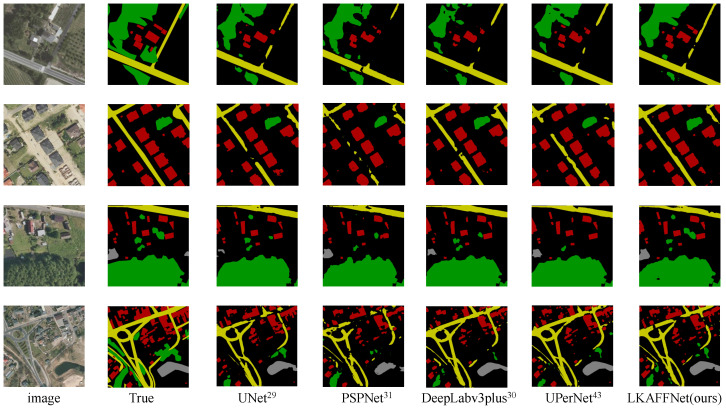
The prediction results of some models. Red represents buildings, green denotes forest, gray indicates water bodies, yellow signifies roads, and black represents the background.

LKAFFNet achieved higher metrics compared to the other models. For a visual comparison, predicted images from UNet [29], PSPNet [31], DeepLabv3+ [30], UPerNet [50], and LKAFFNet are presented in Figure 7.

In the Landcover.ai dataset, urban land types are classified into the following two main categories: buildings and roads. The broad category includes major roads and streets, exhibiting strong internal consistency. However, variations in spectral characteristics due to building shadows at different times can increase internal discrepancies within the road category. Additionally, the presence of trees may interfere with road predictions. The unique characteristics of various building types—residential, commercial, and industrial—further complicate predictions. Therefore, maximizing the use of background features from remote sensing images and integrating contextual semantic information is crucial for improving prediction accuracy in urban land use detection.

Analysis of Table 5 reveals that the HRNet, PSPNet, DenseASPP, and DeepLabv3+ models, as illustrated in Figure 7, exhibit high-performance metrics. Notably, the LKAFFNet model outperforms the others when addressing disturbed areas. For example, in the first row of Figure 7, the shadow of the roadside forest overlaps with the road, resulting in a low recognition rate of the road. Compared with the actual situation, there is a significant difference in the prediction results between other models, and LKAFFNet shows higher accuracy. A similar pattern appears in the third line, where almost all models except LKAFFNet misclassify water bodies.

The high predictive capability of LKAFFNet is attributed to the robust features of LkResNet, which effectively integrates high-dimensional features extracted from the backbone network. The LKAA module merges semantic information across different dimensions, integrating contextual data and distinguishing the contextual relevance of each pixel. The CDFSF module aggregates feature information to guide the reconstruction of low-dimensional features during the feature recovery process. Additionally, the LKAA module dynamically aggregates spatial and channel attention information, enhancing the recovery of edge features for each category.

### 3.5. Generalization Experiment

To assess the generalization performance of LKAFFNet, experiments were conducted using a public dataset of buildings from Wuhan University. This section outlines the details of the experiment.

#### 3.5.1. WHU Building Dataset

The WHU Building Dataset consists of aerial imagery sourced from the New Zealand Land Information Service, featuring vector data for buildings in Christchurch, encompassing approximately 22,000 individual structures. The original ground resolution of the images is 0.075 m. From the original images, 8188 images were cropped, each measuring 512×512 pixels. Among these, 4736 images were randomly selected for the training set, 1036 for the validation set, and 2416 for the test set.

**Figure 8:**
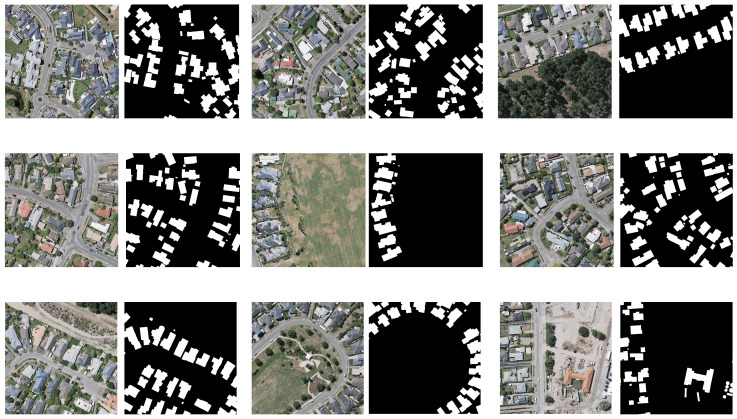
The sample of the WHU Building dataset. Black represents the background; white squares represent buildings.

The land cover types in the WHU Building are divided into background and buildings. Figure 8 shows an example of a WHU Building dataset. In Figure 8, the left side is the original image and the right side is the segmentation result. In the segmentation result, black represents the background and white represents the building.

#### 3.5.2. Results of Generalization Experiments

The experimental conditions for the generalization experiments were consistent with those outlined in Section 3.2. The training results for each model on the land cover dataset are presented in Table 7. As shown in the table, LKAFFNet outperforms the other models in terms of metrics.

**Figure 9:**
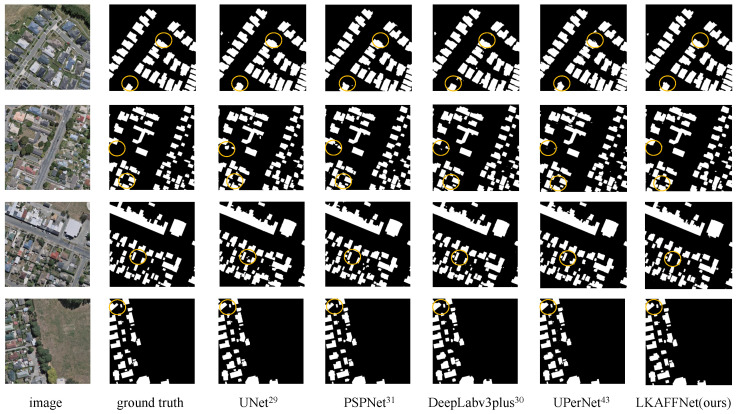
The prediction results of some models on the WHU Building dataset. Black represents the background; white squares represent buildings. The yellow circle represents the contrast part.

Figure 9 displays the prediction results of several models. LKAFFNet demonstrates superior edge prediction when the target objects are relatively small. This indicates that LKAFFNet achieves better segmentation performance compared to the other models.

## 4. Discussion

Accurate urban land use detection is crucial for urban planning and development. This study employs deep learning semantic segmentation techniques to address this challenge. The characteristics of urban land use detection include the following: first, different land use types may interweave and share similar geometric shapes; second, there are significant spectral differences between various land use types. To address these characteristics, we proposed a novel LKAFFNet network architecture.

The advantages of LKAFFNet are evident in several aspects as follows: First, the LKresNet module effectively integrates high-dimensional features extracted from the backbone, facilitating the detection of multi-scale and multi-receptive field input features, which in turn aids in the recovery of low-dimensional features, enhancing the accuracy of potential object clustering and location prediction. Second, the LKAA module consolidates contextual information by merging the features of different resolutions, distinguishing the contextual relevance of each pixel, thereby reducing prediction errors caused by spectral differences within the same object or similarities across different objects. Finally, to bridge the gap between semantic and spatial information, the CDFSF module is designed to dynamically fuse high-dimensional semantic information with low-dimensional spatial information while aggregating edge information for all categories, thereby enhancing the ability to differentiate the geometric features of objects.

Despite LKAFFNet’s strong performance in feature fusion, there remains room for improvement. Future work will focus on enhancing the model’s capability to handle complex categories. For instance, while factories and Central Business Districts (CBDs) both fall under the building category, they exhibit significant differences. We aim to refine the model to more accurately identify distinct functional areas in urban settings, such as industrial zones, residential neighborhoods, and commercial districts.

## 5. Conclusions

In this work, LKAFFNet was developed to achieve state-of-the-art performance in land cover classification. The following three functional modules were designed within LKAFFNet: LkResNet, LKAA, and CDFSF. LkResNet enhances the feature extraction capabilities of the ResNet architecture while reducing computational complexity, thus facilitating better feature fusion. The LKAA module is employed to aggregate spatial and channel attention, while the CDFSF module addresses and fuses multi-scale features.

Through this design, LKAFFNet’s capabilities in land cover classification have been optimized. Experimental comparisons demonstrate that LKAFFNet outperforms previous models on both the LandCover dataset and the WHU Building dataset. Specifically, it achieved a mIoU of 0.8155 on the LandCover dataset and 0.9326 on the WHU Building dataset.

## Figures and Tables

**Table 1:** Experimental results for each group.

Stages (S)/ Layers (L)	Number of Layers	Filter Size, Trend of Changes in the Number of Filters	Activation Function
**S1**	Convolution Layer	3 × 3, same	ReLU
	LKConvolution Layer	Reparameterized Convolution, same	ReLU
	Convolution Layer	3 × 3, 1 × 1, 3 × 3, (same, 2 times, same)	ReLU
**L1**	Convolution Block	1 × 1, Reparameterized Convolution, 3 × 3, 1 × 1, (same, same, 4 times, same)	ReLU
	Identity Block	1 × 1, 4 times	ReLU
**L2**	Convolution Block	1 × 1, Reparameterized Convolution (1/4 times, same)	ReLU
	BN Layer	None	None
	SE Layer	None	Sigmoid
	Convolution Block	1 × 1, 4 times	GELU
	Convolution Block	3 × 3, same	ReLU
	Identity Block	1 × 1, same	ReLU
**L3**	Convolution Block	1 × 1, Reparameterized Convolution (1/4 times, same)	ReLU
	BN Layer	None	None
	SE Layer	None	Sigmoid
	Convolution Block	1 × 1, 2 times	GELU
	Convolution Block	3 × 3, same	ReLU
	Identity Block	1 × 1, 1/2 times	ReLU

**Table 2:** Experimental results for each group (bold represents the best result).

Model	MPA	MIoU	Kappa
LKAFFNet (without Lkresnet)	0.9442	0.7883	0.8953
LKAFFNet (without LKAA)	0.9473	0.7992	0.9009
LKAFFNet (without CDFSF)	0.9459	0.8042	0.8992
LKAFFNet (standard)	**0.9501**	**0.8203**	**0.9069**

**Table 3:** Experimental results for IoU and F1_score by category (bold represents the best result).

Model	IoU by Category						F1_Score by Category					
	Background	Buildings	Woodland	Water	Road	Overall	Background	Buildings	Woodland	Water	Road	Overall
LKAFFNet (without Lkresnet)	0.9157	0.6843	0.878	0.8932	0.5704	0.7883	0.956	0.8125	0.935	0.9436	0.7264	0.8747
LKAFFNet (without LKAA)	0.92	0.7377	0.8823	0.92	0.536	0.7992	0.9583	0.8491	0.9375	0.9583	0.6979	0.8802
LKAFFNet (without CDFSF)	0.9178	0.7249	0.8789	0.9131	0.5861	0.8042	0.9572	0.8405	0.9355	0.9546	0.7391	0.8854
LKAFFNet (standard)	**0.9238**	**0.7496**	**0.8871**	**0.9199**	**0.6213**	**0.8203**	**0.9604**	**0.8569**	**0.9402**	**0.9583**	**0.7664**	**0.8964**

**Table 4:** Experimental results for each group (bold represents the best result).

Model	MPA	MIoU	Kappa
LKAFFNet (without Lkresnet)	0.9721	0.8988	0.9149
LKAFFNet (without LKAA)	0.978	0.9123	0.9192
LKAFFNet (without CDFSF)	0.9745	0.9176	0.9204
LKAFFNet (standard)	**0.9861**	**0.9326**	**0.9287**

**Table 5:** Experimental results of models (bold represents the best result).

Model	MPA	MIoU	Kappa	GFlops	Parameter (M)
DeepLabv3plus [30]	0.9293	0.7213	0.8665	51.5	41.2
DANet [45]	0.9278	0.7272	0.8608	48.5	39.7
HRNet [47]	0.9315	0.742	0.8684	72.8	64.7
OCNet [49]	0.9382	0.7595	0.8836	55.3	50.2
LEDNet [48]	0.926	0.717	0.8587	**9.6**	**4.7**
DenseASPP [46]	0.9415	0.7802	0.8888	28.6	20.3
UPerNet [50]	0.9479	0.8021	0.9024	39.6	31.1
PSPNet [31]	0.9431	0.7647	0.894	45.3	36.9
UNet [29]	0.946	0.8071	0.8983	12.4	7.7
LKAFFNet (ours)	**0.9501**	**0.8203**	**0.9069**	13.5	14.9

**Table 6:** Experimental results for IoU and F1_score by the category of the models (bold represents the best result).

Model	IoU by Category						F1_Score by Category					
	Background	Buildings	Woodland	Water	Road	Overall	Background	Buildings	Woodland	Water	Road	Overall
DeepLabv3plus [30]	0.8947	0.5228	0.8475	0.8764	0.4651	0.7213	0.9444	0.6866	0.9175	0.9341	0.6349	0.8235
DANet [45]	0.8925	0.7036	0.8777	0.6197	0.5425	0.7272	0.9432	0.826	0.9348	0.7652	0.7034	0.8345
HRNet [47]	0.8982	0.6541	0.8515	0.842	0.464	0.742	0.9464	0.7909	0.9198	0.9142	0.6339	0.841
OCNet [49]	0.9069	0.645	0.8704	0.8615	0.5136	0.7595	0.9512	0.7842	0.9307	0.9256	0.6786	0.8541
LEDNet [48]	0.8907	0.5963	0.8455	0.8274	0.425	0.717	0.9422	0.7471	0.9163	0.9055	0.5964	0.8215
DenseASPP [46]	0.9118	0.7253	0.8735	0.8676	0.523	0.7802	0.9539	0.8408	0.9325	0.9291	0.6868	0.8686
UPerNet [50]	0.9212	0.7079	0.8831	0.9184	0.5797	0.8021	0.959	0.829	0.9379	0.9574	0.734	0.8835
PSPNet [31]	0.9143	0.641	0.8803	0.8971	0.4908	0.7647	0.9552	0.7812	0.9363	0.9457	0.6584	0.8554
UNet [29]	0.9181	0.741	0.8765	0.9124	0.5874	0.8071	0.9573	0.8513	0.9342	0.9542	0.7401	0.8874
LKAFFNet (ours)	**0.9238**	**0.7496**	**0.8871**	**0.9199**	**0.6213**	**0.8203**	**0.9604**	**0.8569**	**0.9402**	**0.9583**	**0.7664**	**0.8964**

**Table 7:** Experimental results of models on WHU Building (bold represents the best result).

Model	MPA	MIoU	Kappa
DeepLabv3plus [30]	0.9838	0.9224	0.9171
DANet [45]	0.9617	0.8566	0.8477
HRNet [47]	0.9711	0.8513	0.8485
OCNet [49]	0.9511	0.8726	0.8545
LEDNet [48]	0.9692	0.8618	0.8697
DenseASPP [46]	0.9835	0.9021	0.8958
UPerNet [50]	0.9857	0.9213	0.9273
PSPNet [31]	0.9836	0.9206	0.9153
UNet [29]	0.9724	0.8758	0.8621
LKAFFNet (ours)	**0.9861**	**0.9326**	**0.9287**

## Data Availability

Publicly available datasets were used in this study. The landcover.ai dataset can be found at (https://paperswithcode.com/dataset/landcover-ai, accessed on 1 May 2023). The WHU Building dataset can be found at (http://gpcv.whu.edu.cn/data/building_dataset.html, accessed on 5 January 2022). The code presented in this study is openly available at: (https://github.com/BoChAoChEn1111/LKAFFNet).
